# Studying Electrotaxis in Microfluidic Devices

**DOI:** 10.3390/s17092048

**Published:** 2017-09-07

**Authors:** Yung-Shin Sun

**Affiliations:** Department of Physics, Fu-Jen Catholic University, New Taipei City 24205, Taiwan; 089957@mail.fju.edu.tw; Tel.: +886-2-2905-2585

**Keywords:** electrotaxis, microfluidic chips, cell migration, lab-on-a-chip

## Abstract

Collective cell migration is important in various physiological processes such as morphogenesis, cancer metastasis and cell regeneration. Such migration can be induced and guided by different chemical and physical cues. Electrotaxis, referring to the directional migration of adherent cells under stimulus of electric fields, is believed to be highly involved in the wound-healing process. Electrotactic experiments are conventionally conducted in Petri dishes or cover glasses wherein cells are cultured and electric fields are applied. However, these devices suffer from evaporation of the culture medium, non-uniformity of electric fields and low throughput. To overcome these drawbacks, micro-fabricated devices composed of micro-channels and fluidic components have lately been applied to electrotactic studies. Microfluidic devices are capable of providing cells with a precise micro-environment including pH, nutrition, temperature and various stimuli. Therefore, with the advantages of reduced cell/reagent consumption, reduced Joule heating and uniform and precise electric fields, microfluidic chips are perfect platforms for observing cell migration under applied electric fields. In this paper, I review recent developments in designing and fabricating microfluidic devices for studying electrotaxis, aiming to provide critical updates in this rapidly-growing, interdisciplinary field.

## 1. Introduction

Collective cell movement is involved in various physiological activities including angiogenesis [1,2,3], cancer metastasis [4,5,6], embryonic development [7,8,9] and wound healing [10,11,12]. Different chemical and physical stimuli have been identified and reported as important factors to cause cell migration, phenomena named “-taxis”. Chemotaxis, characterizing how cells move under stimulus of specific chemical gradients, is shown to be crucial in human diseases [13] such as inflammation [14,15], Acquired Immune Deficiency Syndrome (AIDS) [16] and Primary Ciliary Dyskinesia (PCD) [17]. Roussos et al. reviewed in detail how chemotaxis affects various behaviors of tumor cells and stromal cells in vivo [18]. Phototaxis occurs as cells move towards or away from stimulus of light. Lan et al. used a spatial light modulator to create a blue light intensity gradient for inducing directional lung cancer cell migration [19]. Thermotaxis refers to collective cell migration along or opposite to the direction of increasing temperature. Thermotaxis was suggested to be a potential mechanism for navigating mammalian sperm cells [20]. Magnetotaxis describes the behaviors of cells in response to magnetic fields. Magnetotactic bacteria were shown to produce magnetosomes, nanoscale particles acting to align themselves in magnetic fields [21]. Directional cell migration induced by gradients of surface rigidity is termed durotaxis. Well-defined stiffness gradients were generated to study the durotaxis of mesenchymal stem cells, vascular smooth muscle cells, pancreatic stellate cells and fibroblasts [22,23,24,25]. Mechanotaxis is the phenomenon describing how cells migrate via mechanical cues such as fluidic shear stress. When shear stress was applied on mouse micro-vessel endothelial cells, upstream migration with respect to the direction of the flow was observed [26].

Electrotaxis, or galvanotaxis, refers to the directional migration of cells under stimulus of direct current (dc) or alternating current (ac) Electric Fields (EFs). EFs are electric potential gradients and can be generated by applying potential differences across a set of locations. Various cells were reported to migrate collectively under dcEFs. For example, different cell lines of lung cancer were shown to behave differently under dcEF stimuli: CL1-5 cells moved to the anode; A549 cells migrated to the cathode; and CL1-0 cells did not show prominent directional movement [27]. Research conducted by Feng et al. concluded that human Neural Stem Cells (hNSCs) migrated directionally in response to a small EF, and exogenous EFs could be applied to guiding hNSCs toward injured sites in the central nervous system [28]. Under physiologically-relevant dcEFs (100–200 mV/mm), both human peripheral blood lymphocytes and corneal epithelial cells migrated toward the cathode [29,30]. Electrotaxis is also believed to be crucial in promoting wound healing. Early in 1890, du Bois-Reymond first found that an injured finger is electrically positive compared to an intact one. As a wound occurs, an endogenous lateral EF of about 100–200 mV/mm is generated nearby as a result of the breakup of the Transepithelial Potential (TEP) [31,32,33]. Under the stimulus of this EF, cells are able to migrate in a specific direction to repair the damage [34]. Sun et al. developed a novel wound-healing assay for demonstrating that an EF of 150 mV/mm increased the wound-healing rate obviously [35]. A possible reason is that EF induces Reactive Oxygen Species (ROS) production, which in turn promotes cell movement and accelerates wound healing [36,37,38,39,40]. Recently, exogenous electrical stimuli, both dcEFs and acEFs, have been widely applied to wound-healing applications [41,42,43,44]. Using mini pigs as a model, Reger et al. found increased wound-healing rates under both ac- and dc-stimuli when compared to the control wounds [45]. Furthermore, it was demonstrated that dc stimulus reduced wounding area faster than ac, yet ac stimulus decreased wounding volume faster than dc [45]. Bayat et al. concluded that Applying Microampere Electrical Stimulation (MES) daily to skin incision in rabbits significantly accelerated the wound-healing process [46]. The anodal dc stimulation promotes autolysis and re-epithelialization, and the cathodal dc stimulation prevents infection and inflammation [35].

Conventionally, electrotaxis experiments are conducted using Petri dishes or cover glasses wherein cells are cultured and EFs are applied [31,47]. Drawbacks of such simple setups include evaporation of the culture medium, non-uniformity of EFs and low throughput. To overcome these issues, various microfluidic devices have recently been developed for studying electrotaxis in well-controlled micro-environments. Such minimized devices reduce the consumption of cells and reagents, as well as decrease the Joule heating effect [48,49]. Micro-fabricated devices integrated with fluidic systems should provide cells with a stable micro-environment (e.g., temperature, pH, nutrition, stimulus, etc.) where cells remain intact and are stimulated with controllable physical and/or chemical cues. For example, microfluidic chips were used for studying cellular behaviors in response to precise chemical gradients [50,51]. A single-cell migration platform was developed to study single cells’ migration and sort the heterogeneous population on the basis of chemotactic phenotype [52]. In the 1980s and early 1990s, with a clean room facility and technique, microfluidic devices were mostly fabricated on glass and silicon substrates [53,54,55]. In the late 1990s, Polydimethylsiloxane (PDMS) had become a popular material due to its ease of fabrication using soft-lithography [56,57]. Such a material is transparent for easy observation, elastic for minimizing deformation and bio-compatible for culturing cells. More recently, hard materials including Polymethylmethacrylate (PMMA) and Polyethylene Terephthalate (PET) have also been used for rapid prototyping of microfluidic chips with the help of laser ablation and other methods [58,59,60,61]. From glass and silicon to elastomeric materials to compact materials, the cost and time for fabricating microfluidic chips have been greatly reduced.

With advances in techniques of micro-fabrication, microfluidic devices have become more and more popular as a platform for studying electrotaxis of various cells. Advantages of such minimized devices include reduced sample consumption, well-defined EFs, minimized Joule heating, fresh medium supply and increased throughput. In this paper, I review recent developments in microfluidic devices for studying electrotaxis. Traditional devices used for electrotactic studies are noted, and then, microfluidic chips for studying electrotaxis alone or in combination with chemical stimuli are highlighted. This review aims to provide critical and up-to-date research in this rapidly growing, interdisciplinary field.

## 2. Electrotaxis in Traditional Devices

**Figure 1:**
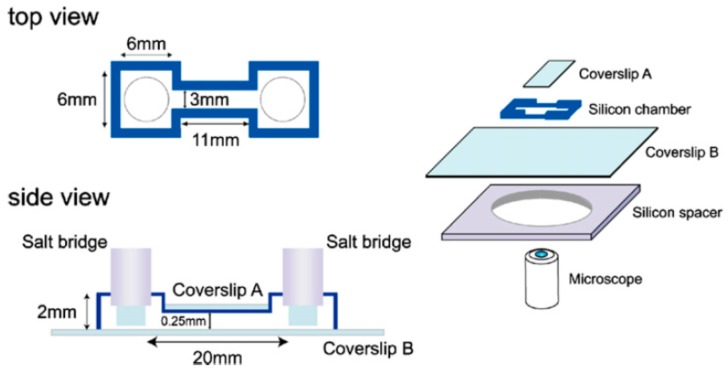
The configuration of the electrotactic chamber used in [49]. Reprinted from Biosystems, 88(3), Masayuki J. Sato, Michihito Ueda, Hiroaki Takagi, Tomonobu M. Watanabe, Toshio Yanagida, and Masahiro Ueda, Input-output relationship in galvanotactic response of Dictyostelium cells, Pages 261–272, Copyright (2007), with permission from Elsevier.

Conventional dish- and cover glass-based devices provide fast and easy electrotactic studies. Djamgoz et al. reported using a galvanotaxis apparatus to study directional migration of rat prostate cancer cells under dcEFs [62]. A glass observation chamber, having a size of 60 mm × 5 mm × 0.2 mm, was made of a sandwich of two glass coverslips. Through Ag/AgCl electrodes immersed in culture medium-filled wells, dc was applied for 6 h to generate EFs of physiological strengths (10–400 mV/mm). The results indicated that rat prostate cancer MAT-LyLu cells (which are highly metastatic) exhibited strong electrotaxis by moving to the cathode, and rat prostate cancer AT-2 cells (which are weakly metastatic) showed no electrotactic behavior. As shown in Figure 1, Sato et al. reported an experimental system for quantitatively analyzing electrotaxis in *Dictyostelium* cells [49]. The electrotactic chamber, having final dimensions of 20 mm × 3 mm × 0.25 mm, was constructed by placing an 18 mm × 10 mm coverslip on top of another 40 mm × 50 mm coverslip using a silicon spacer. To diminish possible byproduct contamination from electrodes, this chamber was connected to Ag/AgCl electrodes, to 2% agar salt bridges and then to a power supply. Cells migrated toward the cathode in a dose-dependent manner with EF strengths of 25–1000 mV/mm.

Another electrotactic chamber of 14.7 mm × 40 mm × 1.5 mm was fabricated by applying a cover glass (20 mm × 40 mm) on a tissue culture plastic dish via sealing with silicone grease [63]. EFs of 50–600 mV/mm were applied to Retinal Pigment Epithelial (RPE) cells through Ag/AgCl electrodes and agar salt bridges. The results showed that RPE cells oriented themselves perpendicular to the EF and migrated toward the anode in an intensity-dependent manner. An electrotactic chamber of 2 cm in length, 1 cm in width and 100 μm in depth was constructed by laminating optically clear acrylic to a culture dish [64]. Murine Adipose-Derived Stromal Cells (mASCs) were cultured inside the chamber and stimulated with EFs of 10–100 mV/mm. These physiological EFs caused cells to move to the cathode in a strength-dependent way and to align themselves perpendicular to the applied field. Song et al. presented protocols of applying EFs to cells cultured in a glass well made of cover slips [47]. The depth of this custom-designed device is adjustable to have room for various samples while providing a stable micro-environment such as temperature, calcium level and pH. It was concluded that EFs, of strengths similar to endogenous ones, act as an important directional cue to induce cell migration during wound healing [65].

**Figure 2:**
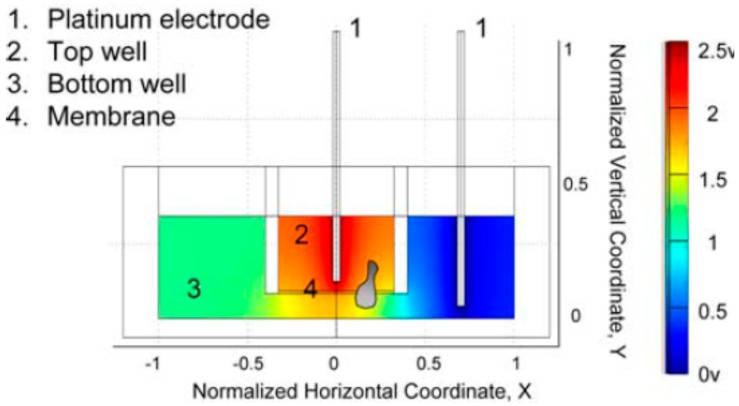
The illustration of the transwell-based electrotactic assay used in [29]. Copyright 2008. The American Association of Immunologists, Inc.

In addition to the above-mentioned dish- and coverslip-based devices, a transwell-based electrotactic assay was reported to have the advantages of ease of operation and high throughput. As shown in Figure 2, the top and bottom wells were loaded with migration medium (RPMI 1640 GlutaMax medium with 10% FBS and 10^6^ human Peripheral Blood Mononuclear Cells (PBMCs) in a 100-μL volume) and medium alone (RPMI 1640 GlutaMax medium with 10% FBS in a 600-μL volume), respectively [29]. To generate an EF, a potential difference of 2.5 V was applied across the transwell by connecting two platinum electrodes, immersed in the top and bottom wells, to a dc power supply. It was found that almost all lymphocyte subsets exhibited increased migration when the cathode was placed in the bottom well, compared with spontaneous migration without EF [29]. With one single transwell plate, this assay is capable of performing up to 24 experiments under different conditions. Although high throughput could be achieved, such a transwell assay is limited to large cell consumption, EF non-uniformity and end-point detection only.

## 3. Electrotaxis in Microfluidic Devices

In a conventional dish-, coverslip- or transwell-based device, cells are cultured in an open, macro-scaled, static (non-flowing) environment. To avoid medium evaporation, reduce cell/reagent consumption and maintain cells in a circulating (flowing) micro-environment, various micro-fabricated devices combined with fluidic components were reported for studying collective cell migration under dcEFs. Such microfluidic devices have closed, well-defined dimensions, providing well-controlled and precise EFs. Furthermore, miniature sizes could increase experimental throughput, as well as reduce Joule heating.

### 3.1. PDMS-Based Microfluidic Devices

Owing to the recent development of the soft-lithography technique, elastomeric materials such as PDMS have been widely applied to fabricating microfluidic devices. In general, to make a PDMS-based microfluidic chip, a SU-8 (an epoxy-based negative photoresist) master mold serves as a patterned template for PDMS casting. After bonding the PDMS piece to a glass slide using oxygen plasma treatment, micro-channels with dimensions of a few tens to a few thousands of micrometers are formed. Rezai et al. fabricated a PDMS-based electrotactic chamber for studying electrotaxis of *Caenorhabditis elegans* (*C. elegans*) [66]. The system included four parts: (1) a micro-channel made of one patterned PDMS piece and one plane PDMS piece (size: 80 μm in depth and 5 cm in length with a variable width); (2) an integrated unit including syringe pumps, inlet and outlet connections for manipulating worms; (3) an actuator consisting of a power supply and electrodes; (4) a monitoring unit composed of a microscope and a camera. By punching through the PDMS piece, copper electrodes were inserted into the reservoirs. With various widths from 2 mm–150 μm, this micro-channel could generate EFs of 100–1200 mV/mm. It was found that animals of different stages responded differently to the same EF range: the L3 stage worms responded to EFs above 400 mV/mm with migration speeds of 100–216 μm/s; the L4 stage worms responded to EFs of 400–1000 mV/mm with speeds of 220–340 μm/s; the young adults responded to EFs of 200–400 mV/mm with speeds of 296–471 μm/s [66]. This device could serve as a high-throughput platform for applications in drug screening using *C. elegans* as the model.

**Figure 3:**
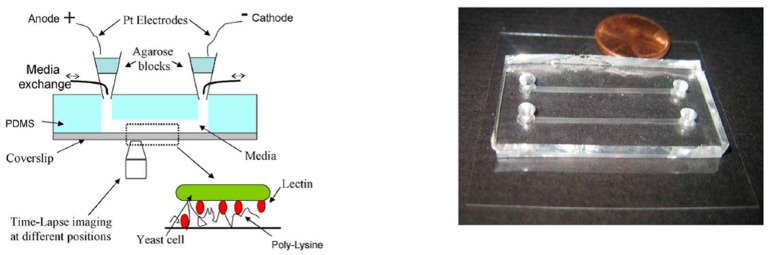
The configuration (**left**) and picture (**right**) of the PDMS-based electrotactic chamber used in [67]. Reprinted from Current Biology, 20(8), Nicolas Minc and Fred Chang, Electrical Control of Cell Polarization in the Fission Yeast *Schizosaccharomyces pombe*, Pages 710–716, Copyright (2010), with permission from Elsevier.

As shown in Figure 3, Minc et al. constructed a micro-channel of 200 μm high, 500 μm wide and 4 cm long in PDMS to electrically control cell polarization of the fission yeast *Schizosaccharomyces pombe* (*S. pombe*) [67]. Via oxygen plasma treatment, the patterned PDMS piece was bonded to a coverslip coated with polylysine. Lectin was subsequently coated on the surface for effective capture of yeasts. Pipette tips served as reservoirs allowing for medium exchange in the channel, and agarose blocks were placed on top of the reservoirs to prevent cells from potentially toxic products releasing from the electrodes. Platinum electrodes placed inside the reservoirs were connected to a power supply for generating an EF of 5000 mV/mm. Under the stimulus of exogenous EF, these *S. pombe* cells were found to orient themselves orthogonally to the field [67]. Li et al. developed a similar micro-device for observing the electrotactic responses of T lymphocytes [68]. A PDMS micro-channel of 1 cm × 350 μm × 100 μm in size and a glass slide were brought together to create an irreversible seal after treating with air plasma. Medium reservoirs were created by punching out two wells at the ends of the channel, and two platinum electrodes placed inside the reservoirs were connected to a dc power supply for generating an EF of 700 mV/mm across the micro-channel. The experimental results showed that activated human peripheral blood T cells moved to the cathode under this applied EF.

Zhao et al. presented a miniature, independently operable microfluidic platform, named ElectroTaxis-on-a-Chip (ETC), for high-throughput quantitative electrotactic studies [69]. This microfluidic system integrated Ag/AgCl electrodes, lithium battery cells, an electricity regulator and a miniature LED voltmeter into one compact, credit card-sized device. This ETC device consisted of three PDMS pieces. The top and middle layers had wells holding the batteries, a resistor, a voltmeter and a switch. The electrical wires and the EF gradient generator were embedded in the middle and bottom layers, respectively. Two reservoirs connecting the EF generator and electrodes were placed over the micro-channel inlet and outlet. By using the R-2R resistor ladder structure, an infinitely expandable EF gradient generator was designed and fabricated for studying EF-stimulated cell movement in a high-throughput manner. With repeating identical units of one series resistor of R and one shunt resistor of 2R, this module was expandable to generate as many EF strengths as possible. This ETC device was applied to studying the effective range of EF strength causing electrotactic behaviors of human corneal epithelial cells (hTCEpi cells). An ETC device having an 11-level resistor ladder generated an EF gradient from 2.1 mV/mm–1.6 V/mm. The results indicated that hTCEpi cells moved to the cathode in response to EFs in an intensity-dependent way [69].

Recently, Li et al. reported two PDMS microfluidic chambers for long-term electrotaxis-based heterogeneity study of lung cancer cells [70]. In the first device, by plasma-bonding the PDMS piece to a glass slide, two parallel channels with dimensions of 4 mm (length) × 30 μm (height) × 300, 150, and 100 μm (width) were integrated into one single chip. An inlet for cell loading, an outlet and two wells for electrodes were punched through the PDMS layer. With three different widths, this chip could generate EFs of four different intensities including 0 mV/mm as a control. It was shown that lung adenocarcinoma H1975 cells migrated cathodally with varying cellular orientation, and the migration speed increased with increasing EF strength [70]. A second chamber composed of a cell immobilization structure, an EF generator and a cell retrieval unit was used for further studies of electrotactic heterogeneity. This device consisted of two PDMS layers molded from the Printed Circuit Board (PCB) masters etched by FeCl3 solution [71]. The top and bottom layers had heights of 30 and 9 μm, respectively, and these two pieces were bonded together via plasma treatment. Small micro-channels with a 9-μm depth were formed, allowing attached, but not suspended cells to move through. By using this device, cells with strong and weak electrotactic responses could be collected separately for further processes. It was concluded that in the absence of EF, H1975 cell motility was associated with Epidermal Growth Factor Receptor (EGFR) expression, while under EF stimulus, it was related to PTEN expression [70]. Various PDMS-based micro-devices have also been developed for studying the electrotactic responses of *C. elegans* [72,73,74], parasitic nematodes [73], activated T cells [75], bovine meniscus cells [76], mouse fibroblasts [77], human prostate cancer PC3 cells [77] and mammary epithelial cells [78].

### 3.2. PMMA-Based Microfluidic Devices

Compared to PDMS-based devices, laser writing of hard materials such as PMMA for the fabrication of microfluidic chips provides a lower cost and easier manufacturing process. Therefore, PMMA-based microfluidic devices are more applicable for conducting electrotactic studies in a time-saving and high-throughput manner. The time for fabricating a PMMA microfluidic chip is usually less than 10 min, while the time for constructing a PDMS-based one takes longer than 12 h. To investigate the electrotactic behaviors of human Peripheral Blood Leukocytes (PBLs), Lin et al. developed a microfluidic device in PMMA substrates, which included laser-cut micro-channels of 1.5 cm long, 500 μm wide and 100 μm deep [29]. This electrotactic chamber consisted of one glass slide (75 mm × 25 mm × 1 mm, serving as the bottom of the microfluidic device), one Melinex sheet with adhesive on both sides (100-μm thick, serving as the sidewalls of the micro-channel), one Melinex sheet with no adhesive (50-μm thick, used to seal the channel) and two 200-μL pipette tips (serving as medium reservoirs). Two platinum electrodes placed inside the medium reservoirs were connected to a power supply for generating an EF of 100 mV/mm. The results indicated that human PBLs moved to the cathode in a dcEF with a strength similar to that observed in vivo.

**Figure 4:**
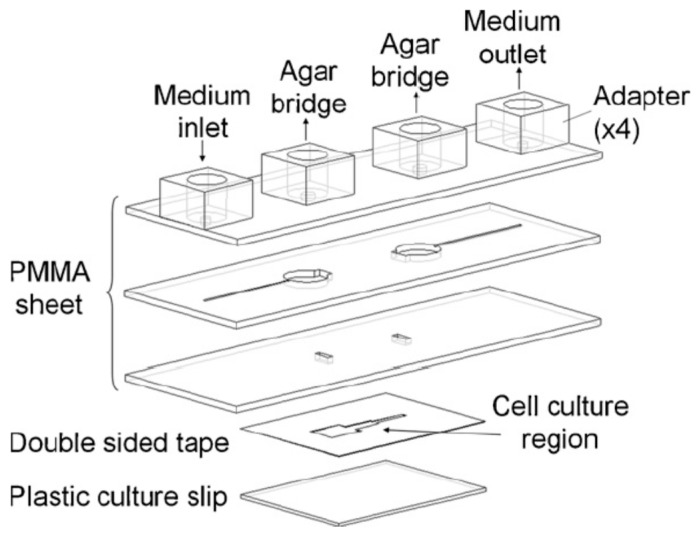
The configuration of the PMMA-based Multi-Field Chip (MFC) used in [79]. Reprinted from Biosensors and Bioelectronics, 24(12), Ching-Wen Huang, Ji-Yen Cheng, Meng-Hua Yen, and Tai-Horng Young, Electrotaxis of lung cancer cells in a multiple-electric-field chip, Pages 3510–3516, Copyright (2009), with permission from Elsevier.

By using rapid laser ablation, Huang et al. fabricated a PMMA-based Multi-Field Chip (MFC) for long-term electrotactic studies of lung cancer cells [79]. A CO_2_ laser scriber was employed to write specific patterns on PMMA sheets and double-sided tapes [80,81]. As illustrated in Figure 4, this MFC was composed of three thermally-bonded PMMA sheets, one plastic culture slip and one double-sided tape bonded to the slip and the bottom PMMA piece. A micro-channel in the double-sided tape had dimensions of 24 mm (length) × 70 μm (height) × 5, 1.67 and 1 mm (width) and served for cell culture and EF generation. Four adapters were attached to the top PMMA sheet with two serving as the medium inlet/outlet and the other two connecting to agar salt bridges. The entire electrotactic system consisted of an MFC, a customized ITO heating chip, a syringe pump, an X-Y motor stage, two agar salt bridges, Ag/AgCl electrodes, a power supply, an ammeter and a digital camera-equipped, inverted microscope. It is worth noting that a transparent ITO heating chip was stacked beneath the MFC to ensure temperature homogeneity over the entire chip [82]. With an applied voltage of 10 V, EF strengths of 74, 205 and 331 mV/mm were generated in three segments of the cell culture area. It was observed that lung cancer CL1-5 cells moved to the anode, yet CL1-0 cells showed no clear electrotactic behaviors [79]. Furthermore, after 1 h of EF stimulus, CL1-5, but not CL1-0 cells tended to align themselves perpendicular to the applied EF [79]. To clarify the transcriptional mechanisms of the electrotaxis of CL1-5 cells, another Large Electric-Field Chip (LEFC) was developed [83]. Cells cultured on this chip were treated with an EF of 300 mV/mm for 2 h and then harvested for microarray analysis. This LEFC chip consisted of three layers of PMMA sheets, a commercial 15-cm cell culture dish and a layer of double-sided tape bonded to the dish and the bottom PMMA piece. Compared with the MFC, the cell culture channel of this LEFC had much larger dimensions of 24 mm (width) × 75 mm (length) × 70 μm (height) for collecting as many cells as possible. The results indicated that various EF-regulated genes were closely related to telomerase RNA component gene regulation, adherens junction and tight junction [83]. Moreover, many significantly regulated genes encoding cell membrane proteins were observed, indicating that dcEF could affect the activity of these proteins in signal transduction [83].

Soon after, the same group developed a new microfluidic device named the Multiple electric Field with Uniform Flow chip (MFUF chip) for providing multiple EF strengths to cells under a uniform flow field [84]. Similarly, this MFUF chip consisted of three thermally-bonded PMMA pieces, a cell dish and a double-sided tape bonded to the dish and the bottom PMMA sheet. The micro-channel in the double-sided tape was specially designed so that four EF strengths with a ratio of 7.9:2.8:1:0 were obtained in four cell culture segments having an identical cross-sectional area and thus a uniform flow. Such a uniform flow is important in electrotactic studies since variations in flow velocity and shear stress have been reported to influence cell morphology and alignment [85,86,87]. The performance of this MFUF chip was first validated by studying the electrotactic responses of lung cancer CL1-0 and CL1-5 cells, showing similar results to what were reported earlier in the MFC chip [79,84]. Then, the electrotaxis of human oral squamous cell carcinoma HSC-3 cells was studied, showing that HSC-3 cells exhibited weaker electrotactic responses, but higher motility than CL1-5 cells [84]. To study the EGFR and Receptor Tyrosine Kinase (RTK) signaling in the electrotactic behaviors of lung adenocarcinoma CL1-0 and CL1-5 cells, a PMMA chip termed XLEFC (eXtra Large Electric Field Chip) was designed and fabricated for providing a uniform dcEF stimulus to a large amount of cells [88]. This XLEFC consisted of a PMMA assembly, a cell culture assembly and a 15-cm cell culture dish. The PMMA assembly included three layers of PMMA sheets. The top layer had medium inlet/outlet and adapters for agar salt bridges. The middle layer contained two current rectifying chambers for passing the current from the slat bridges, through the slits in the bottom layer, to the cell culture area. With coefficients of variation of 1.2% from simulation and 2.3% from measurement, a uniformly-distributed EF was generated across the cell culture region. The cell culture assembly, having dimensions of 100 mm × 69 mm × 0.6 mm (L × W × H), was composed of one soft-acrylic sheet, two fluoroplastic tapes and two double-sided tapes. The culture area of this XLEFC was claimed to have a six-fold increase compared to conventional devices [88]. After EF stimulation, protein phosphorylation of CL1 cells was characterized using commercial antibody arrays and Western blotting. The results showed that the phosphorylation profiles in dcEF-treated cells were different from those in EGF-treated cells, excluding EGFR-triggered pathways in CL1 cells’ electrotactic responses [88].

**Figure 5:**
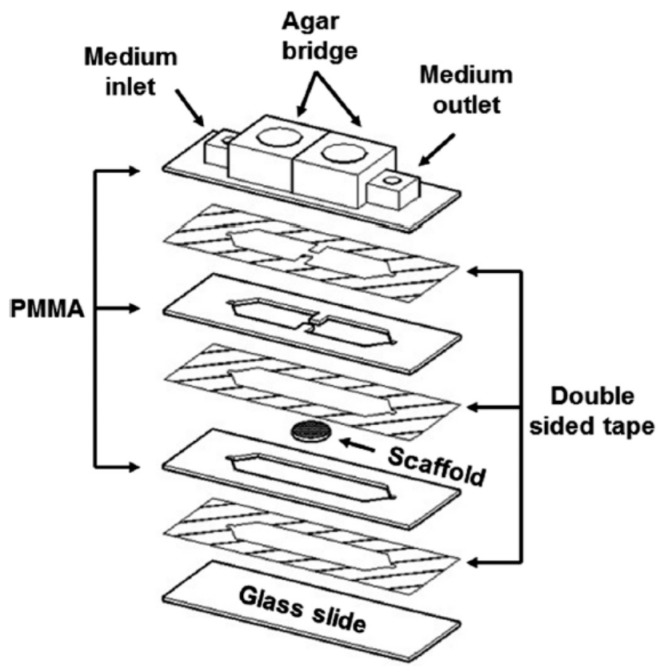
The configuration of the electrotactic system used in [27]. Reprinted from Sun, Y.S.; Peng, S.W.; Lin, K.H.; Cheng, J.Y. Electrotaxis of lung cancer cells in ordered three-dimensional scaffolds. *Biomicrofluidics* **2012**, *6*, 014102, with the permission of AIP Publishing.

Wang et al. reported combing a PMMA-based Micro-fluidic Electric-field Cell-culture (MEC) chip with Structured-Illumination Nano-Profilometry (SINAP) for quantitative studies of cancer-cell filopodium growth under dcEFs [89]. Four layers of this MEC chip included, from bottom to top, one patterned PMMA sheet, one glass culture slide, one double-sided tape and one cover glass. The micro-channel in the double-sided tape had dimensions of 15 mm × 4 mm × 70 μm (L × W × H) and served as the cell culture chamber. Two agar salt bridges immersed in Phosphate-Buffered Saline (PBS) reservoirs were connected to the chip, and Ag/AgCl electrodes placed inside the same PBS reservoirs were connected to a dc power supply. By using the super-resolution SINAP [90], filopodia of lung cancer CL1-5 cells were observed to prefer growing on the side facing the cathode under EFs of 180–250 mV/mm [89]. Furthermore, from confocal fluorescence microscopy images, this cathodally-biased filopodium growth was found to be related to the distribution of EGFRs [89]. To study electrotaxis in three-Dimensional (3D) micro-environments, Sun et al. incorporated 3D cell culture scaffolds with a PMMA-based microfluidic device [27]. 3D scaffolds with ordered arrays of uniform spherical pores were fabricated using a PDMS-based flow-focusing device. Images of confocal fluorescence microscopy indicated that the sizes of top-to-bottom and side-to-side connected pores were around 50 and 25 μm, respectively. As shown in Figure 5, in the microfluidic system, three PMMA sheets were bonded together via two double-sided tapes, and then, this PMMA assembly was bonded to a glass slide via another double-sided tape. Two protrudent pieces of the middle PMMA layer were used to clip the scaffold and prevent it from sliding away during observation. To perform electrotactic experiments, a cell suspension at a density of 5 × 10^6^–10^7^ cells/mL was pipetted into the scaffold for incubation in 5% CO_2_ at 37 °C for at least 2 h. Then, the scaffold was transferred to the microfluidic system where a dcEF of 338 mV/mm was applied to cells within the scaffold. By calculating the ellipticity, defined as [(long axis)-(short axis)]/(long axis) for an ellipse-shaped cell, cell morphology of various lung cancer cells cultured in both 3D and two-Dimensional (2D) micro-environments was investigated. It was observed that CL1-5 and A549, but not CL1-0 cells preferred round-shaped morphology when cultured in 3D scaffolds, in contrast to those having an ellipse-shaped morphology in 2D substrates [27]. Under EF stimulation, CL1-5 cells moved to the anode, A549 cells migrated to the cathode and CL1-0 cells showed no clear electrotactic response. Furthermore, compared to 2D cells, 3D cells showed much weaker electrotactic responses mainly because steric hindrance caused some cells to be blocked by the walls of the pores [27].

**Figure 6:**
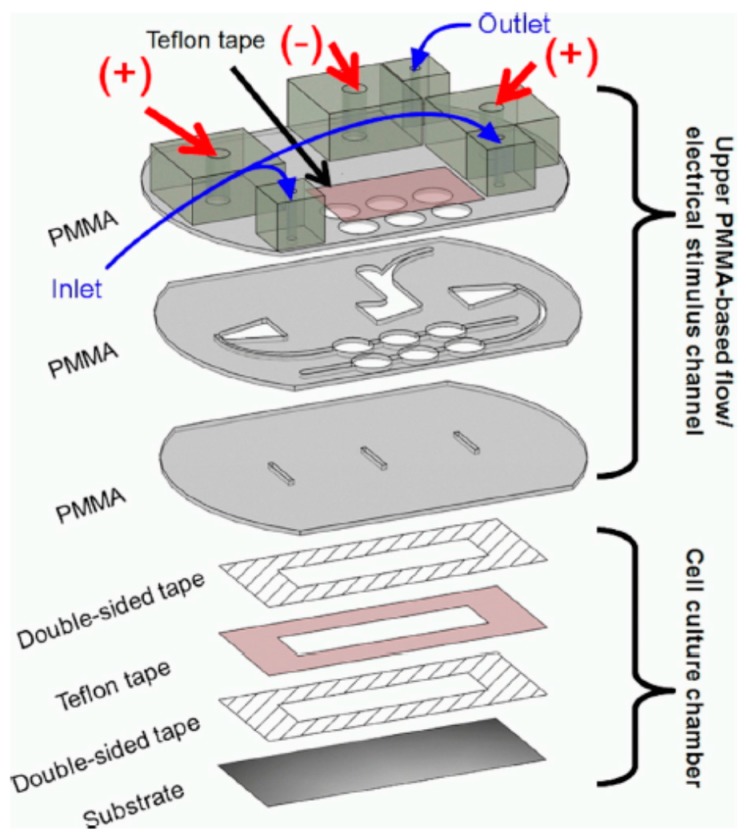
The configuration of the Electrically-Stimulated Wound-Healing Chip (ESWHC) used in [35]. Reprinted from Sun, Y.S.; Peng, S.W.; Cheng, J.Y. In vitro electrical-stimulated wound-healing chip for studying electric field-assisted wound-healing process. *Biomicrofluidics* **2012**, *6*, 34117, with the permission of AIP Publishing.

**Figure 7:**
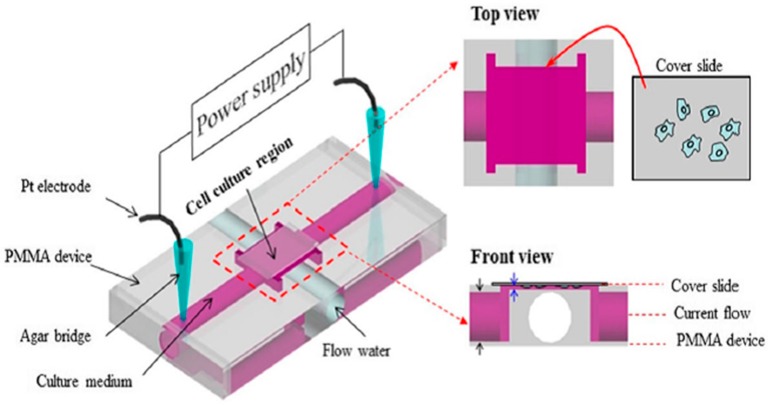
The configuration of the PMMA-based device used in [91]. Analytical and Bioanalytical Chemistry, Effects of direct current electric fields on lung cancer cell electrotaxis in a PMMA-based microfluidic device, 2017, 409(8), Pages 2163–2178, Yaping Li, Tao Xu, Xiaomei Chen, Shin Lin, Michael Cho, Dong Sun, and Mengsu Yang. With permission of Springer.

Since EF is believed to be related to wound healing, Sun et al. reported a microfluidic Electrically-Stimulated Wound-Healing Chip (ESWHC) combining EF with a modified barrier assay [35]. This device was capable of providing an exogenous EF best mimicking the endogenous one for studying wound-healing processes. As illustrated in Figure 6, this ESWHC consisted of a top PMMA-based flow/EF chamber and a bottom cell culture chamber. Three PMMA substrates were thermally bonded together to form the top assembly. Six holes created on the top and middle PMMA pieces and covered with a Teflon tape served as bubble trappers. The middle PMMA layer was patterned to form micro-channels for both medium flow and electrical conduction (via ion flow). Three slits on the bottom PMMA piece were used for both medium and ion flowing into the underneath cell culture region. The cell culture chamber was composed of one Teflon tape, two double-sided tapes and one culture dish. To create a wound, a piece of tape (width = 0.6 mm) was attached to the center of the cell culture chamber, and then, this tape-made barrier was torn off after a fibroblast monolayer was formed. The middle slit in the bottom PMMA piece was connected to a AgCl electrode as the cathode, and the outside two slits were connected to Ag electrodes as the anode. Under such a configuration, an EF flowing to the wounding site was generated. This ESWHC was used to study the dependence of wound-healing rate on EF, serum and wound-healing-promoting drugs. The results showed that: (1) both serum and EF increased the wound-healing rate obviously; (2) a low concentration of β-lapachone (a wound-healing-promoting drug) in the presence of EF increased the healing rate, but an overdose of β-lapachone combined with EF decreased the rate instead [35]. This ESWHC was claimed to provide an easy, fast and high-throughput platform for in vitro drug screening before conducting in vivo trials. Recently, Li et al. reported a PMMA microfluidic chip for electrotactic studies of various Non-Small Cell Lung Cancer (NSCLC) cell lines [91]. As shown in Figure 7, this PMMA-based device was composed of two channels for medium and water flow, a groove of 465 μm in depth and 18 mm in width and a glass slide of 24 mm × 30 mm in size. After culturing cells on the glass slide for 48–60 h, this slide was placed upside down facing the groove for generating a dc through the medium channel. The water channel beneath the slide could efficiently dissipate Joule heat to avoid over temperature. Under physiological dcEFs (200–600 mV/mm), it was observed that: (1) for adenocarcinoma cells, H1975 cells exhibited cathodal directionality with increased motility, while HCC827 cells moved slightly to the anode with reorientation and increased motility; (2) for large cell carcinoma cells, H460 cells only exhibited cathodal directionality under the stimulus of dcEF, while H1299 cells moved to the anode in a dose-dependent manner with reorientation and increased motility [91]. Moreover, both Ca^2+^ influx via activated stretch-activated calcium channels and Ca^2+^ release from intracellular storage were found to be related to the electrotactic behaviors of various lung cancer cells [91].

## 4. Combination of Electrotaxis and Chemical Stimuli

In vivo, cells are subjected to various physiological stimuli including physical and chemical ones. For example, chemical gradients of different cytokines and dcEFs were reported to occur concurrently around in vivo cancer cells [92]. Several receptor tyrosine kinases such as Vascular Endothelial Growth Factor Receptor (VEGFR) and EGFR were shown to be associated with electrotaxis [88,93]. Therefore, it is important to be able to elucidate the underlying mechanisms of chemical-involved electrotactic behaviors. Micro-fabricated devices capable of generating controllable, precise EFs and chemicals provide a platform for conducting such studies. Li et al. designed and fabricated a PDMS-based microfluidic chip that can produce controllable single or coexisting chemical and electrical stimuli [75]. By using contact photolithography, a 100 μm-thick SU-8 master was first patterned on a silicon substrate. Then, the PDMS replica was fabricated and bonded to a glass slide via oxygen plasma. One outlet well at one end of the main channel (350 μm (W) × 1 cm (L)) and two inlet wells at the other end of the channel were generated. Two electrode wells connecting two side channels were generated, filled with medium and connected to a dc power supply via two platinum electrodes. These two side channels were parallel to the main channel and connected to it by 40 thin channels (20 on each side). By infusing medium and chemokine solutions into the device from two fluidic inlets, a chemical concentration gradient could be generated in parallel to the applied dcEF. This device was used to study the movement of activated human peripheral blood T cells in single or coexisting chemokine CCL19 gradients and exogenous dcEFs. T cells were shown to migrate toward a chemokine CCL19 gradient of 100 nM and move to the cathode of an applied dcEF of 50 mV/mm [75]. Under coexisting CCL19 gradient and dcEF, the cathodal migration of T cells exhibited a reduced orientation index, but the migration speed was not affected compared to that in the single CCL19 gradient or single dcEF [75]. Later, Kao et al. presented another PDMS microfluidic chip for regulating chemotaxis of lung cancer cells via dcEFs [92]. By using soft-lithography, a thin PDMS channel with dimensions of 20 μm (H) × 0.2 mm (W) and two thick PDMS channels with dimensions of 100 μm (H) × 1 mm (W) were fabricated and bonded to a glass slide. The thin channel was perpendicular to two thick channels with an intersected length of 0.5 mm. After connecting the two ends of the thin channel to a dc power supply via two agar salt bridges, a dcEF was generated along the 0.5-mm length. By injecting medium and chemical into the chip from two thick channels, a chemical concentration gradient was achieved also along the 0.5-mm length of the thin channel. Under the stimulation of EGF chemical gradients, lung cancer CL1-5 cells showed increased directedness with increasing concentration gradient, but the migration speed seemed to reach a maximum of 0.2 μm/min [92]. Furthermore, it was observed that both the directedness and the migration speed increased with increasing dcEF from 0–540 mV/mm [92]. Combing chemotaxis and electrotaxis, it was found that a EGF gradient of 0.5 μM/mm could nearly be balanced by a dcEF of 360 mV/mm [92]. Compared with the device reported earlier by Li et al., the present chip was claimed to have advantages of better EF homogeneity, smaller flow fields in the observation region, improved temperature homogeneity and structural simplicity [75,92].

**Figure 8:**
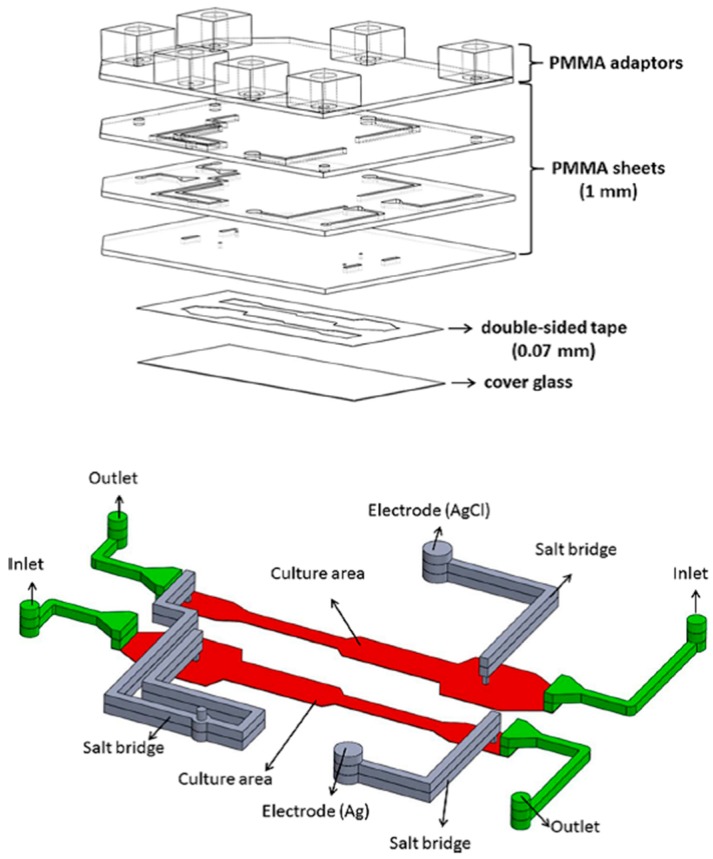
The configuration (top) and micro-channel (bottom) of the PMMA-based microfluidic chip used in [36]. Reprinted from Wu, S.Y.; Hou, H.S.; Sun, Y.S.; Cheng, J.Y.; Lo, K.Y. Correlation between cell migration and reactive oxygen species under electric field stimulation. *Biomicrofluidics* **2015**, *9*, 054120, with the permission of AIP Publishing.

**Figure 9:**
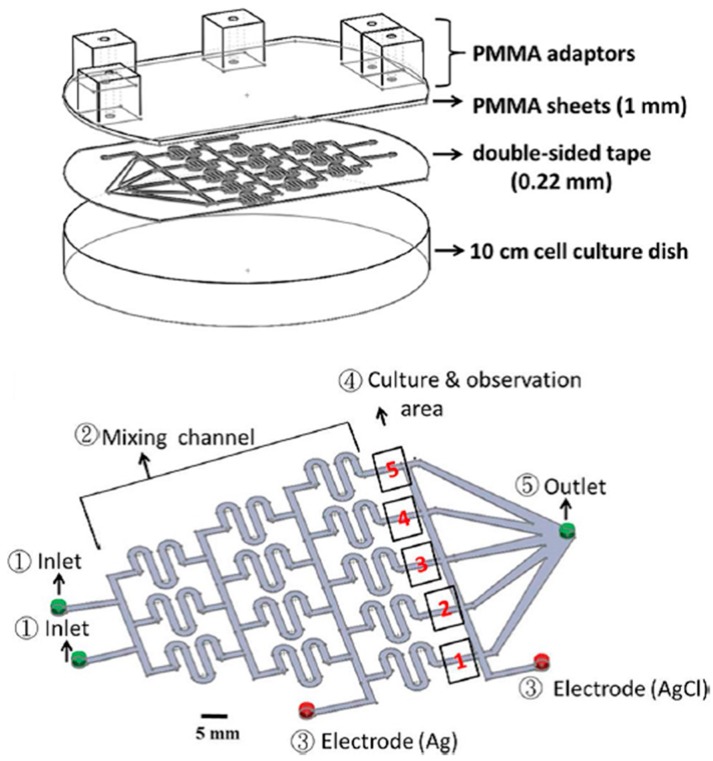
The configuration (top) and micro-channel (bottom) of the PMMA-based microfluidic chip used in [94]. Microfluids and Nanofluids, A microfluidic device for studying the production of reactive oxygen species and the migration in lung cancer cells under single or coexisting chemical/electrical stimulation, 2016, 20(1), 11 pages, Kai-Yin Lo, Shang-Ying Wu, and Yung-Shin Sun. With permission of Springer.

To study the molecular mechanisms of electrotaxis, Hou et al. developed a Multichannel Dual electric Field (MDF) chip for investigating the chemical-modulated electrotactic responses of lung cancer cells [93]. This device could provide eight combined chemical/electrical stimuli in one single experiment. This MDF chip consisted of four layers of PMMA substrates thermally bonded together, one double-sided tape and one cover glass. The first PMMA layer had adapters for medium inlets/outlets and agar salt bridges. The other three PMMA layers provided networks for ion flow (for electrical conduction) and medium flow (for cell culture). In the patterned double-sided tape, four micro-channels having dimensions of 0.07 mm (H) × 3 mm (W) × 46 mm (L) served as cell culture areas, with each of them having its own inlet and outlet. By positioning the anode in one end of the micro-channel and placing the cathode in the middle and the other end of the micro-channel, one EF region and one control region were generated inside the cell culture area. This device could therefore generate eight different combinations of electrical/chemical stimulations simultaneously (4 chemical × 2 electrical). Electrotaxis of lung cancer CL1-5 cells was investigated using this MDF chip. The results indicated that both the migration speed and the anodal directedness of CL1-5 cells were reduced after cells were treated with Phosphatidylinositide 3-Kinase (PI3K) blocker (LY294002) [93]. Moreover, the directedness, but not the migration speed, was affected after suppressing Rho-associated Coiled-coil Kinase (ROCK) in the EF-stimulated CL1-5 cells by Y27632, a ROCK inhibitor [93]. This chip can provide a rapid, high-throughput platform for electrotactic studies under single or combining chemical and electrical stimuli. Soon after, a PMMA-based microfluidic device which could generate four different EF intensities in combination with two different chemicals was developed [36]. The correlation between cell migration and ROS production under EF stimulation was investigated. As shown in Figure 8, four layers of PMMA sheets were thermally bonded together and then attached to a culture dish by using a double-sided tape. In the patterned double-sided tape (height = 70 μm), two micro-channels having widths of 4, 2.07, 4.14 and 8.28 mm served as cell culture areas, with each of them having its own inlet and outlet. By applying a potential difference across the micro-channel, four different EF strengths were generated inside the cell culture region. This device could therefore generate eight different combinations of electrical/chemical stimulations simultaneously (2 chemical × 4 electrical). NIH 3T3 fibroblasts cultured inside this chip were stimulated with combined EFs and chemicals to study their migration and ROS production. It was found that after treating cells with EF in the presence or absence of β-lapachone, both the migration speed and the ROS production increased with increasing EF strength [36]. A good linear correlation between cell migration speed and ROS production suggested that EF and β-lapachone enhance cell migration via intermediate ROS production [89]. In addition, an antioxidant, α-tocopherol, was observed to diminish ROS production, causing a decreasing migration speed [36]. Lo et al. designed and fabricated another PMMA microfluidic chip for investigating the migration and ROS production of lung cancer cells under single or combining chemical and electrical stimuli [94]. As illustrated in Figure 9, this device was simply composed of one PMMA sheet, one double-sided tape and one 10-cm culture dish. A typical Christmas tree structure with two inlets was patterned in the double-side tape, providing five relative concentrations of 0, 1/8, 1/2, 7/8 and 1 in the cell culture areas. Furthermore, by considering all fluidic segments as resistors with resistance proportional to their lengths, five different EF strengths (0.49*I*, 0.25*I*, 0.13*I*, 0.08*I* and 0.05*I*, where *I* is the applied dc) were generated inside these culture areas. The results indicated that: (1) the production of ROS increased as the concentration of H_2_O_2_ increased from 0–200 μM; (2) the formation of ROS increased as the EF strength increased, but less ROS was produced after adding honokiol; (3) cell migration speed increased as the EF strength increased, but the speed decreased after adding honokiol [94].

## 5. Conclusions

Table 1 lists the advantages and drawbacks of conventional and microfluidic devices used in electrotactic studies. Although conventional dish- and coverslip-based devices provide easy observation and observable cell migration, they are limited by bad EF control and low throughput. The transwell-based device can perform experiments in a high-throughput manner, but it has drawbacks of bad EF control, large cell consumption and end-point observation only. Micro-fabricated devices integrated with fluidic systems overcome these problems by generating controllable and precise EFs, reducing Joule heating, reducing cell consumption, providing direct cell observation and providing a high-throughput platform. Especially for PMMA-based microfluidic devices, simple and inexpensive fabrication makes them perfect tools for rapid electrotactic experiments. Methods and protocols for the design and fabrication of PMMA microfluidic chips are detailed and visualized in [95,96].

Despite more and more successful applications of microfluidic devices in electrotactic studies, there are still further improvements that need to be discussed. First, for the purposes of time-saving and screening-based studies, a high-throughput platform is highly desirable. This can possibly be done by further minimizing and automatizing the microfluidic system. Second, it will be of great interest to do single-cell analysis by combining the microfluidic device with super-resolution microscopies such as vibrational microscopy, confocal microscopy, phase microscopy and fluorescence correction/lifetime microscopy. Such applications have been reported in [89,97]. Thirdly, since in vivo cells are surrounded by various extracellular matrices, it is relevant to study the electrotaxis of cells cultured in a 3D micro-environment. As reported, electrotactic responses of lung cancer cells in ordered 3D scaffolds have been investigated [27]. However, better control over chemical or electrical stimuli inside these scaffolds is required. Finally, on the basis of successful electrotactic studies, microfluidic devices can be designed for clinical applications such as biosensors, Circulating Tumor Cell (CTC) chips and non-invasive therapeutic devices. For example, electrical stimuli are known to manipulate immune cells, as well as interrupt cancer metastasis, providing potential therapeutic approaches in treatments of immune and cancerous diseases. As micro-fabrication techniques such as soft-lithography and laser ablation become more and more improved and mature, microfluidic devices have provided biologists and biochemists a platform for investigating the physiological roles and mechanisms of electrotaxis in a more efficient way.

## Figures and Tables

**Table 1:** Electrotaxis in conventional and microfluidic devices. EF, Electric Field.

		Advantages	Drawbacks	References
**Conventional Devices**	**Dish-based or coverslip-based**	Easy operation Observable cell migration	Bad EF control Low throughput	[47,49,62,63,64]
	**Transwell-based**	Easy operation High throughput	Bad EF control Large cell input End-point observation only	[29]
**Microfluidic Devices**	**PDMS-based**	Controllable and precise EFs Reduced Joule heating High throughput Observable cell migration Low cell/reagent consumption	Complicated fabrication	[66,67,68,69,70,72,73,74,75,76,77,78]
	**PMMA-based**	Controllable and precise EFs Simple and inexpensive fabrication Reduced Joule heating High throughput Observable cell migration Low cell/reagent consumption	Possible toxic by-products	[27,29,35,79,83,84,88,89,91]
